# MicroRNA‐1275 induces radiosensitization in oesophageal cancer by regulating epithelial‐to‐mesenchymal transition via Wnt/β‐catenin pathway

**DOI:** 10.1111/jcmm.14784

**Published:** 2019-11-15

**Authors:** Congying Xie, Youyi Wu, Zhenghua Fei, Ya Fang, Shenlan Xiao, Huafang Su

**Affiliations:** ^1^ Department of Radiotherapy and Chemotherapy The First Affiliated Hospital of Wenzhou Medical University Wenzhou China; ^2^ Departments of Radiation Oncology The Third Affiliated Hospital of Wenzhou Medical University Ruian China

**Keywords:** epithelial‐mesenchymal transition, miR‐1275, oesophageal cancer, radioresistance, Wnt/β‐catenin

## Abstract

Acquired radioresistance is one of the main obstacles for the anti‐tumour efficacy of radiotherapy in oesophageal cancer (EC). Recent studies have proposed microRNAs (miRNAs) as important participators in the development of radioresistance in various cancers. Here, we investigated the role of miR‐1275 in acquired radioresistance and epithelial‐mesenchymal transition (EMT) in EC. Firstly, a radioresistant cell line KYSE‐150R was established, with an interesting discovery was observed that miR‐1275 was down‐regulated in KYSE‐150R cells compared to the parental cells. Functionally, miR‐1275 inhibition elevated radioresistance in KYSE‐150 cells via promoting EMT, whereas enforced expression of miR‐1275 increased radiosensitivity in KYSE‐150R cells by inhibiting EMT. Mechanically, we demonstrated that miR‐1275 directly targeted WNT1 and therefore inactivated Wnt/β‐catenin signalling pathway in EC cells. Furthermore, WNT1 depletion countervailed the promoting effect of miR‐1275 suppression on KYSE‐150 cell radioresistance through hampering EMT, whereas WNT1 overexpression rescued miR‐1275 up‐regulation‐impaired EMT to reduce the sensitivity of KYSE‐150R cells to radiation. Collectively, our findings suggested that miR‐1275 suppressed EMT to encourage radiosensitivity in EC cells via targeting WNT1‐activated Wnt/β‐catenin signalling, providing a new therapeutic outlet for overcoming radioresistance of patients with EC.

## 1 INTRODUCTION

Oesophageal cancer (EC) is one of the most common and incurable malignancies worldwide.^1^, ^2^ Although therapeutic strategies for EC have been largely improved,^3^, ^4^, ^5^ the 5‐year survival of patients is still disappointing, which mainly results from increased resistance to the chemotherapy, radiotherapy or chemoradiotherapy in EC patients at more advanced stages.^6^ Thus, to overcome chemoresistance and radioresistance, studies aimed to understand underlying molecular mechanism are greatly needed.

MicroRNAs (miRNAs) are a class of small non‐coding RNAs that regulate gene expression at post‐transcriptional level.^7^ Recently, researchers have increasingly paid attention to the parts of miRNAs in modulating cellular biological events in human diseases including cancer.^8^ Importantly, miRNAs have been lately demonstrated to be involved in the development of chemoresistance or radioresistance. For example, down‐regulated miR‐199a‐3p in hepatocellular carcinoma results in the increased doxorubicin sensitivity.^9^ miR‐494‐3p enhances radiosensitivity in human oral squamous carcinoma cells.^10^ MiR‐1275 is a newly recognized miRNA which plays a paradoxical role in different human cancers. For instance, miR‐1275 hinders tumour growth in hepatocellular carcinoma by targeting the oncogenic IGF2BPs.^11^ Conversely, miR‐1275 promotes the progression in head and neck squamous cell carcinoma through up‐regulating IGF‐1R and CCR7.^12^ Importantly, the down‐regulation of miR‐1275 in radioresistant EC cells has already been proven previously.^13^ However, the precise function of miR‐1275 in EC remains to be identified.

Epithelial‐mesenchymal transition (EMT) is a process that primarily describes tissue remodelling and cell migration during embryonic development to shape future organisms. Over the years, this process has also been discovered in disease course, such as tumour progression.^14^ A growing number of studies explained that EMT is largely implicated in radioresistance of numerous cancers due to the close association with the plastic phenotype of cancer stem cells (CSCs).^15^, ^16^ Recently, reports have lately suggested that ionizing radiation induces transforming growth factor beta (TGF‐β), while TGF‐β facilitates EMT in human mammary cells.^17^, ^18^ Intriguingly, EMT induced by ionizing radiation in turn promotes radiation resistance.^19^ Also, mounting evidence has unearthed that miRNAs have important functions in regulating EMT,^20^ such as miR‐200 family, miR‐194, miR‐192 family and so on.^21^, ^22^, ^23^ Nevertheless, the relationships among miR‐1275, EMT and radioresistance in EC have never been probed yet.

Currently, we aimed to investigate the role and potential mechanism of miR‐1275 in the acquired radioresistance of EC cells.

## 2 MATERIALS AND METHODS

### 2.1 Cell culture

The cell line of human oesophageal squamous carcinoma cell line KYSE‐150 was purchased from the American Type Culture Collection and the radioresistant subtype KYSE‐150R cells were established as described.^24^ Then, both the KYSE‐150 and KYSE‐150R cells were grown in RPMI‐1640 (Gibco, Life Technologies Inc) supplemented with 10% foetal bovine serum (FBS; HyClone, South Logan), 100 U/mL penicillin and 100 μg/mL streptomycin (Invitrogen) and stored in a humidified atmosphere at 37°C, containing 5% CO_2_.

### 2.2 Establishment of radioresistant cell line KYSE‐150R

The radioresistant KYSE‐150R cell line was developed as previously described.^24^ Briefly, 1 × 10^6^ of KYSE‐150 cells suspended in 100 μL culture medium were seeded in the culture flasks and cultured for 2 days, followed by irradiation treatment with 1 Gy of X‐ray at a dose rate of 100 cGy/min through a high energy linear accelerator (Varian 600C, USA). After irradiation, the culture medium was immediately renewed and the cells were further incubated until reached approximately 90% confluence. Subsequently, cells were subcultured into new flasks after trypsinization and collection, followed by irradiation again (second fraction) once they reached nearly 50% confluence. One and a half months later, the radioresistant cell populations were obtained when treated with a total doses of 21 Gy after repeated above procedures (1 Gy 3 times, 2 Gy 3 times and 4 Gy 3 times) for 12 times, which were proved to maintain a radioresistant phenotype for at least 5 months of 50 passages. And such irradiated cells were not used in the experiments until at least 1 month after the last exposure. At the meantime, the parental KYSE‐150 cells were also passaged and cultured under the same conditions except irradiation all the time.

### 2.3 Cell transfection

Cell transfection was performed using Lipofectamine 2000 (Invitrogen) in light of the manufacturer's instructions. The miR‐1275 inhibitor and negative control (NC inhibitor) oligonucleotides, the miR‐1275 mimic and the negative control (NC mimic) oligonucleotides, and the small interfering RNA (siRNA) oligonucleotides targeting WNT1 mRNA (si‐WNT1) and the negative control (si‐NC) were obtained from GenePharma Corporation. Meanwhile, the full‐length WNT1 cDNA was subcloned into pcDNA3.1 vector (Invitrogen) for WNT1 overexpression (pcDNA3.1/WNT1) with the empty vector as negative control. After planted and grown for 1 day in 6‐well plates, KYSE‐150 and KYSE‐150R cells were transfected appropriately with the plasmids obtained above. Transfection for 48 hours later, the cells were collected and maintained for following use. The transfection efficiency was confirmed by quantitative real‐time PCR.

### 2.4 Quantitative real‐time PCR (qRT‐PCR)

Based on the manufacturer's instructions, qRT‐PCR analyses were carried out for evaluation of the expression of miR‐1275 and its target gene WNT1. Total RNA was extracted from KYSE‐150 and KYSE‐150R cells by the use of Trizol reagent (Invitrogen) followed by reverse transcription into cDNA using Reverse Transcription Kit (Takara) or TaqMan miRNA Reverse Transcription Kit (Applied Biosystems). Thereafter, qRT‐PCR analyses were conducted using SYBR‐Green Real‐Time PCR Kit (Takara). U6 snRNA and GAPDH served as normalized controls for miR‐1275 and WNT1 mRNA, respectively. And relative expression of genes was calculated by using 2^−ΔΔCt^ method. All experiments were performed in triplicate. The primer sequences were shown as below: miR‐1275, forward: 5′‐TGGGGGAGAGGCTGTC‐3′, reverse: 5′‐GAACATGTCTGCGTATCTC‐3′; WNT1, forward: 5′‐CTCTTCGGCAAGATCGTCAACC‐3′, reverse: 5′‐CGATGGAACCTTCTGAGCAGGA‐3′; U6, forward: 5′‐CTCGCTTCGGCAGCACAT‐3′, reverse: 5′‐TTTGCGTGTCATCCTTGCG‐3′; GAPDH, forward: 5′‐GTCTCCTCTGACTTCAACAGCG‐3′, reverse: 5′‐ACCACCCTGTTGCTGTAGCCAA‐3′.

### 2.5 Irradiation and colony formation assays

Cells were processed with X irradiation at indicated doses (0, 2, 4, 6 and 8 Gy). Dosimetry showed a central dose rate of 4 Gy/min. After the irradiation treatments, the irradiated cells and a group of negative control without irradiation were stored at 37℃ in humidified air with 5% CO_2_ for 14 days of incubation for detecting colony formation activity in vitro. After washed twice with PBS, cells were fixed with methanol for 5 minutes and stained with 0.1% crystal violet (Beyotime Institute of Biotechnology) for 3 minutes at 37°C. The amount of colonies containing more than 50 cells was counted and fitted into a single‐hit multi‐target model using GraphPad Prism 6.0. Thereafter, the radiosensitization survival curves of the cells were depicted accordingly, with several parameters including Dq and D0 value were calculated and shown in Table 1. The plating efficiencies (PE) and the survival fractions (SF) were, respectively, measured as well according to the following equations: PE = number of colonies/number of cells seeded × 100%; SF = number of colonies after irradiation/number of cells seeded × (1/PE). The sensitization enhancement ratio (SER) was measured as the D0 values of relative control group divided by D0 value of the corresponding treated group. All the experiments referred were performed in triplicate.

**Table 1:** Radiation survival curve parameters for KYSE150 and KYSE150R under diverse conditions

Cell lines	Transfection	D0 (Gy)	Dq (Gy)
KYSE150	Blank	2.23 ± 0.09	2.83 ± 0.05
	NC inhibitor	2.19 ± 0.06	2.78 ± 0.03
	miR‐1275 inhibitor	2.86 ± 0.11	3.46 ± 0.13
	miR‐1275 inhibitor + si‐WNT1	2.07 ± 0.13	2.81 ± 0.04
KYSE150R	Blank	2.75 ± 0.23	3.17 ± 0.04
	NC mimic	2.79 ± 0.20	3.32 ± 0.12
	miR‐1275 mimic	2.28 ± 0.25	3.12 ± 0.22
	miR‐1275 mimic + pcDNA3.1/WNT1	2.92 ± 0.16	3.58 ± 0.15

Note

D0 value means the mean lethal dose, and Dq means the dose quasithreshold.

### 2.6 MTT assay

MTT assay was performed to test cell viability in vitro. In short, cells were seeded in a 96‐well plate and incubated for 24, 48, 72 and 96 hours at 37°C in humidified atmosphere with 5% CO_2_. After the addition of 20 μL 5 mg/mL MTT [(3‐(4,5‐dimethylthiazol‐2‐yl)‐2,5‐diphenyltetrazolium bromide)] solution into each well, the plates were continuously incubated at 37°C for 4 hours. Subsequently, the supernatant was removed and 100 µL dimethyl sulfoxide (DMSO) was supplemented to dissolve remaining formazan for 30 minutes. Finally, the value of optical density (OD) at 490 nm was examined with the microplate reader ELx808 (Bio‐Tek Instruments Inc). All the experiments were conducted in triplicate.

### 2.7 Transwell assay

Transwell assay was performed using 8‐μm transwell inserts (Millipore, USA) to evaluate cell migration capacity in vitro. The cells (5 × 10^4^ of each kind of cells) suspended in serum‐free medium were placed in the upper chambers with the lower chambers supplemented fully with medium containing 10% FBS as the chemoattractant. After being cultured for 24 hours, cells migrating to the lower chambers were fixated with methanol and stained using 0.1% crystal violet. The number of migrated cells in 5 random views was counted under the inverted microscope (Olympus). All the experiments were conducted in triplicate.

### 2.8 Wound‐healing assay

Wound‐healing assay was also performed to assess cell migration in vitro. The cells were seeded and cultured in 6‐well plates until 80% confluence. Afterwards, a wound among the cells was created artificially using the tip of a sterile 200‐μL pipette tube. Then, cells were washed and further grown in medium with 2% FBS. The wound closure was observed regularly and imaged under a microscope 36 hours later. The migration rate was determined as the ratio of healed wound to the wound line. All the experiments were conducted in triplicate.

### 2.9 Western blot

Total proteins from KYSE‐150 and KYSE‐150R cells were isolated with radioimmunoprecipitation assay (RIPA) buffer (Pierce) containing protease inhibitor. Then, protein concentration was measured with BCA™ Protein Assay Kit (Pierce). After that, cell protein with equal quantity was separated using 10% sodium dodecyl sulphate polyacrylamide gel electrophoresis (SDS‐PAGE) and then transferred to polyvinylidene difluoride (PVDF) membranes (EMD Millipore). The membranes were then blocked with 5% skim milk followed by incubation overnight with primary antibodies at 4℃. After being washed for three times with Tris‐buffered saline and Polysorbate 20 (TBST) buffer, the membranes were further incubated with the secondary antibodies at 37°C for 2 hour. At length, the protein bands were viewed using ChemiDoc XRS System (Bio‐Rad). The primary antibodies involved in were as follow: WNT1 (ab15251), E‐cadherin (ab40772), N‐cadherin (ab18203), Vimentin (ab92547), β‐catenin (ab32572), p‐β‐catenin (ab27798), c‐myc (ab32072), Slug (ab51772) (above all from Abcam) as well as GSK‐3β (#12456), p‐GSK‐3β (#9323) and GAPDH (#5174) (from Cell Signaling Technology). GAPDH acted as a loading control.

### 2.10 Luciferase reporter assay

Luciferase reporter assay was performed to detect the relationship between WNT1 and miR‐1275. WNT1 containing the predicted binding site (WNT1‐WT) or the mutant binding site (WNT1‐Mut) was amplified and cloned into pGL3 vector (Promega), respectively. MiR‐1275 inhibitor, NC inhibitor, miR‐1275 mimic or NC mimic was co‐transfected into HEK‐293T cells with pGL3‐WNT1‐WT or pGL3‐WNT1‐Mut. After 48 hours of transfection, the luciferase activity was determined through Dual‐Luciferase Reporter Assay System (Promega). All the experiments were conducted in triplicate.

### 2.11 RNA pull‐down assay

First of all, the biotin‐labelled RNAs including Bio‐miR‐1275‐WT, Bio‐miR‐1275‐Mut and negative control (Bio‐NC) were obtained with Biotin RNA Labelling Mix (Roche). Meanwhile, cells were processed in a lysis buffer (Gibco) and cell lysates were incubated with the biotinylated RNAs overnight. Subsequently, the magnetic beads with streptavidin (Gibco) were added and co‐incubated for 48 hours. After being washed, the bonding RNAs in the beads were eluted down and then detected by qRT‐PCR analysis. All the experiments were repeated for three times.

### 2.12 RNA immunoprecipitation (RIP) assay

RIP assay was performed to verify the interaction between WNT1 and miR‐1275 in vitro, using the Magna RNA‐binding protein immunoprecipitation kit (Millipore) according to the manufacturer's instructions. The cells were lysed and incubated overnight in RIP buffer containing magnetic beads coated with anti‐Ago2 antibody or anti‐IgG (negative control). After being washed, the RNAs deposited on beads were eluted and determined using qRT‐PCR. All the experiments were repeated for three times.

### 2.13 TOP/FOP flash assay

TOP/FOP flash assay was conducted to assess the activity of Wnt/β‐catenin signalling pathway by using the Dual‐Luciferase Reporter Assay Kit (Promega) according to the instruction provided by the manufacturer. The TOP/FOP flash reporter plasmids with wild‐type (CCTTTGATC; TOP flash) or mutated (CCTTTGGCC; FOP flash) T cell factor/lymphoid enhancer‐binding factor (TCF/LEF) binding sites were purchased from Upstate Biotechnology (Waltham, Massachusetts, USA). Then, the reporter plasmids and pRL‐TK Renilla plasmid were co‐transfected into KYSE‐150 cells with miR‐1275 inhibitor or NC inhibitor and KYSE‐150R cells with miR‐1275 mimic or NC mimic, under the use of Lipofectamine 2000 (Invitrogen) in accordance with the manufacturer's protocol. Luciferase signals including Firefly and Renilla luciferase activity were measured 48 hours after transfection with the renilla luciferase activity as a normalized control for the firefly luciferase activity. All the experiments were repeated for three times.

### 2.14 In vivo xenograft experiment

In vivo experiment was performed under the experimental animal use guidelines of the ethics committee of The First Affiliated Hospital of Wenzhou Medical University. A total of 20 male BALB/c nude mice (aged 6 weeks) were purchased from Model Animal Research Center of Nanjing University (Nanjing, China) and then randomly classified into four groups (with 5 mice each group). Then, half of the mice were subcutaneously inoculated with KYSE‐150 cells transfected with miR‐1275 inhibitor while another half with NC inhibitor‐transfected KYSE‐150 cells. When tumours grew to about 8.0 mm (range from 7.7 to 8.2 mm) in diameter, the mice in indicated group initiated to undergo radiation treatment by use of a small‐animal irradiator (Co‐V, Theratron 780; MDS Nordion) with a cobalt‐60 source. Subsequently, mice mechanically immobilized in a jig were exposed to a single dose of 5 Gy at a dose rate of 0.955 Gy/min. The measurements of tumour growth were taken every 4 day. Tumour volumes were assessed using the formula V = 1/2 × L × W^2^ (L, the longest dimension; W, the shortest dimension). After 28 days following the injection, the mice were sacrificed and tumour weights were measured. Then, tumours were embedded in paraffin and sliced for further assay. All the experiments were repeated for three times.

### 2.15 TUNEL assay

The paraffin‐embedded sections were applied to assess the apoptosis rate of tumours obtained above by detecting apoptotic DNA strand breaks using a TUNEL Staining Kit (Roche Inc) following the manufacturer's protocol. Then, cells were stained with DAPI (a cell nucleus marker; Invitrogen). The number of TUNEL‐ and DAPI‐positive nuclei was calculated after the mages captured by an EVOS fluorescent microscope (AMG). And cell apoptosis was expressed as the ratio of TUNEL‐positive nuclei to DAPI‐positive nuclei (total nuclei).

### 2.16 Immunohistochemical (IHC) staining

Immunohistochemistry (IHC) assay was conducted on the paraffin‐embedded sections obtained in in vivo experiments. In brief, the sections were incubated with primary antibodies against Ki67 (1:100; Roche) and then stained by a universal DAB detection kit (Roche). Then, the images of stained sections were obtained using a light microscope, followed by the counting of the Ki67‐positive cells in five random fields.

### 2.17 Statistical analyses

The data were analysed using GraphPad Prism 6 and then showed as the mean ± standard deviation (SD). Differences were evaluated by a two‐tailed unpaired Student's *t* test between two groups or a one‐way analysis of variance among at least three groups. Statistical significance was identified as *P* < .05.

## 3 RESULTS

### 3.1 MiR‐1275 was significantly down‐regulated in radioresistant EC cells

**Figure 1:**
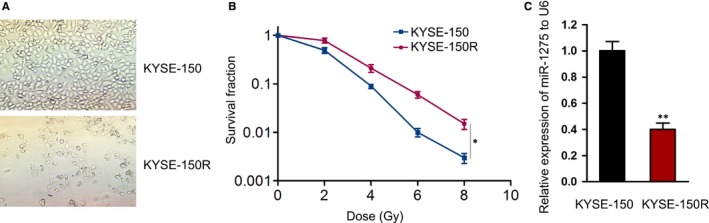
MiR‐1275 was down‐regulated in KYSE‐150R cells. A, Representative images of KYSE‐150 and KYSE‐150R cells captured under a light microscope. B, The survival rate of KYSE‐150 and KYSE‐150R cells after being exposed to radiation at specific doses (0, 2, 4, 6 and 8 Gy) was assessed by colony formation assays. C, Relative expression of miR‐1275 to U6 in KYSE‐150 and KYSE‐150R cells was detected via qRT‐PCR. *P < .05, **P < .01

To evaluate the effect of miR‐1275 on the sensitivity of EC cells to radiotherapy, we established a radioresistant EC cell line KYSE‐150R which showed limited response to irradiation treatment as described.^24^ Seen from Figure 1A, the radioresistant KYSE‐150R cells were morphologically changed into spindle shapes (more elongated) with weakened adhesion and strengthened motility. In addition, the survival fraction of KYSE‐150R cells was identified to be noticeably higher than the parental KYSE‐150 cells (Figure 1B) and the SER for KYSE150R cells was 0.811, corroborating that KYSE‐150R cells became less sensitive to irradiation treatment. Finally, we detected the expression of miR‐1275 in KYSE‐150R and its parental cells. As a result, the level of miR‐1275 was dramatically reduced in KYSE‐150R cells in contrast to that in KYSE‐150 cells (Figure 1C). Collectively, we speculated that the expression level of miR‐1275 might be strongly associated with the radiosensitivity of EC cells.

### 3.2 MiR‐1275 elevated radiosensitivity of EC cells by inhibiting EMT

**Figure 2:**
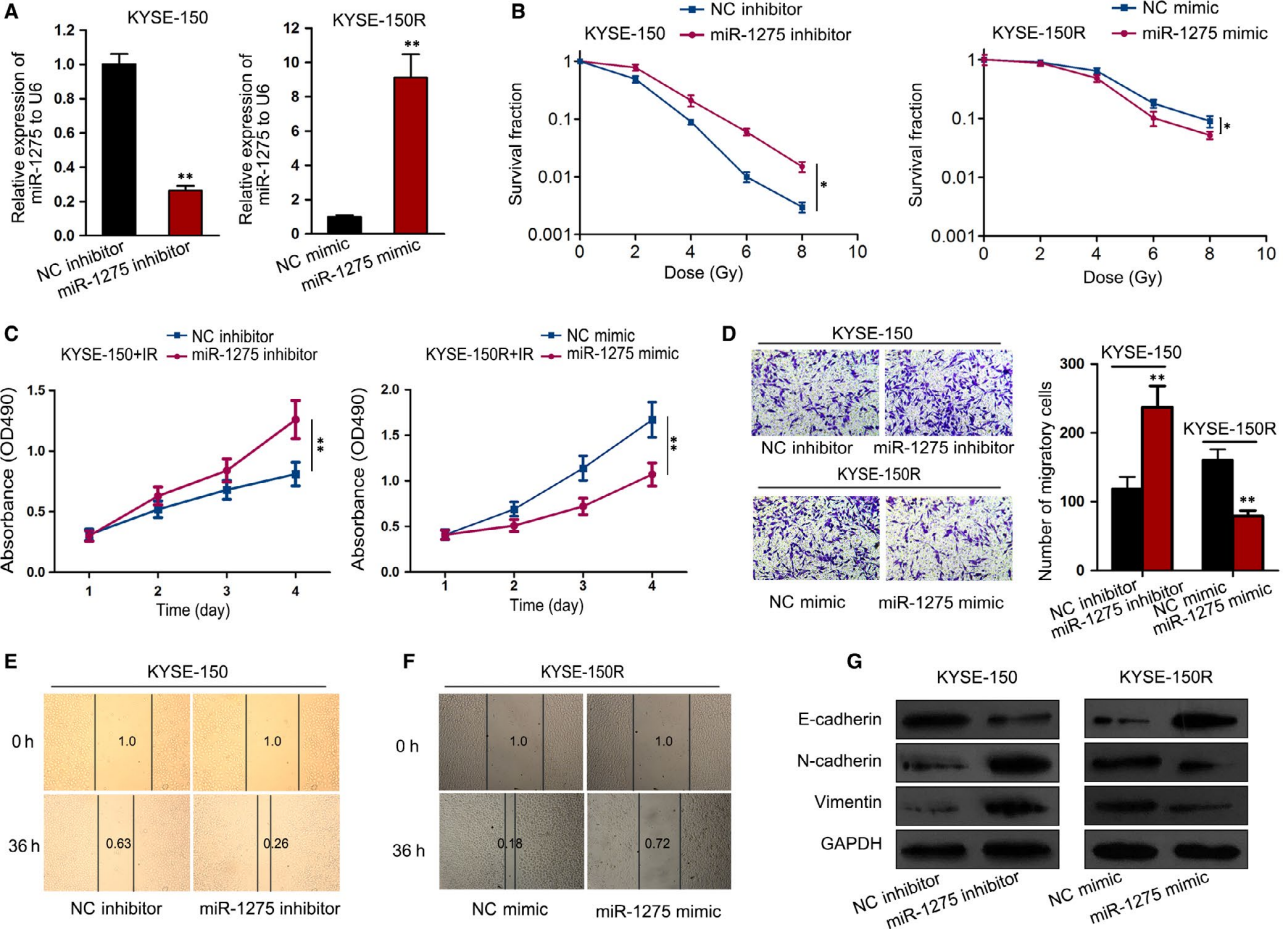
MiR‐1275 promoted radiosensitivity by inhibited EMT in EC cells. KYSE‐150 cells transfected with miR‐1275 inhibitor or NC inhibitor and KYSE‐150R cells transfected with miR‐1275 mimic or NC mimic were used for subsequent assays. A, Relative expression of miR‐1275 in above cells was examined via qRT‐PCR. B, The survival fraction of cells was tested by colony formation assays after exposed to radiation at doses of 0, 2, 4, 6 and 8 Gy. C, The viability of indicated cells with irradiation treatment of 6 Gy was determined by MTT assay. D‐F, Cell migration capacity of KYSE‐150 cells and KYSE‐150R cells was evaluated using Transwell assay (D) and wound‐healing assay (E, F). G, The levels of EMT‐associated proteins in above EC cells were estimated by Western blotting. *P < .05, **P < .01

To specifically probe the role of miR‐1275 in the development of radioresistance in EC cells, loss‐ and gain‐of‐function assays were, respectively, conducted in KYSE‐150 cells and KYSE‐150R cells. First of all, we confirmed the decreased expression of miR‐1275 in KYSE‐150 cells after being transfected with miR‐1275 inhibitor and the increased miR‐1275 level in KYSE‐150R cells under the transfection of miR‐1275 mimic (Figure 2A). Subsequently, the results of colony formation assay elucidated that miR‐1275 inhibition resulted in elevated radioresistance whereas miR‐1275 up‐regulation led to increased radiosensitivity (Figure 2B). Besides, the SER was 0.766 for miR‐1275‐inhibited KYSE150 cells and 1.224 for miR‐1275‐up‐regulated KYSE150R cells. Additionally, the irradiated KYSE‐150 cells seemed to survive better under miR‐1275 suppression while KYSE‐150R cells with enhanced miR‐1275 expression exhibited a confined proliferative ability upon IR treatment (Figure 2C). Based on the above results, miR‐1275 was greatly indicated to contribute to the sensitivity of EC cells to radiation exposure.

Previously, a report revealed that more than one‐third of EC patients cannot be cured by CCRT treatment because of distant metastasis.^25^ Besides, epithelial‐mesenchymal transition (EMT) is a hallmark of tumour metastasis^26^ and a process largely related to cancer radioresistance.^27^ Thus, we assumed that miR‐1275 influenced the radiosensitivity of EC cells through effecting EMT. Firstly, we assessed whether miR‐1275 had an impact on EC cell migration that can be enhanced during EMT.^26^ As a result, Transwell assay suggested that inhibition of miR‐1275 distinctly augmented the number of migratory KYSE‐150 cells while its overexpression apparently lessened the migration ability of KYSE‐150R cells (Figure 2D). Accordantly, the result of wound‐healing assay also showed the repressive impact of miR‐1275 on cell migration of EC cells (Figure 2E,F). Furthermore, we proved that the epithelial marker E‐cadherin was down‐regulated while the mesenchymal markers including N‐cadherin and Vimentin were up‐regulated in miR‐1275‐repressed KYSE‐150 cells, whereas the opposite outcomes were detected in KYSE‐150R cells in response to the transfection of miR‐1275 mimic (Figure 2G). Altogether, miR‐1275 reduced radioresistance of EC cells by suppressing EMT.

### 3.3 MiR‐1275 directly targeted WNT1 and inactivated Wnt/β‐catenin pathway

**Figure 3:**
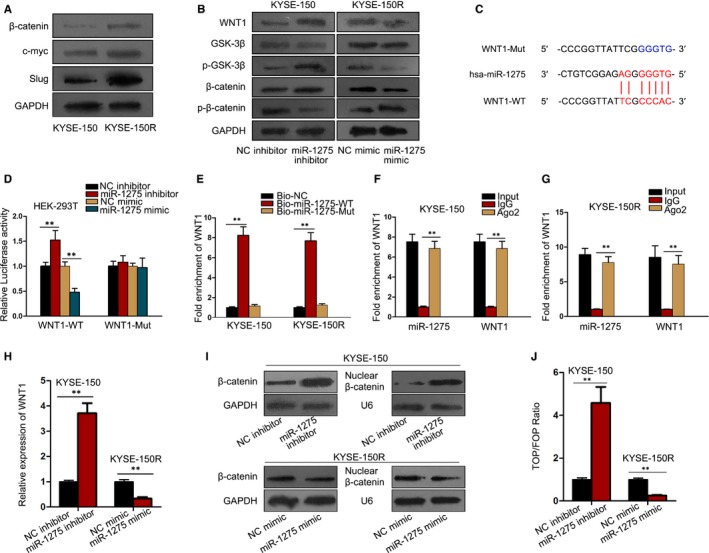
MiR‐1275 inactivated Wnt/β‐catenin pathway in EC cells through directly targeting WNT1. A, Western blot results of the protein level of β‐catenin and its targets in KYSE‐150 and KYSE‐150R cells. B, The protein levels of the members involved in Wnt/β‐catenin signalling in miR‐1275‐inhibited KYSE‐150 cells and miR‐1275‐up‐regulated KYSE‐150R cells were examined by Western blot. C, Predicted binding sites between miR‐1275 and WNT1, as well as the sequences of WNT1‐Mut with mutated binding sites in 3′‐UTR of WNT1. D, Luciferase reporter assays for the luciferase activity of WNT1‐WT and WNT1‐MUT in HEK‐293T cells with different transfections. E‐G, RNA pull‐down assay (E) and RIP experiments (F, G) were conducted in indicated EC cells for further confirmation of the interaction between miR‐1275 and WNT1. H, The effect of miR‐1275 on the expression of WNT1 in EC cells was assessed by qRT‐PCR. I, The level of total β‐catenin and nuclear β‐catenin in KYSE‐150 cells with miR‐1275 inhibition and KYSE‐150R cells with ectopic expression of miR‐1275 was detected via Western blot. J, TOP/FOP flash assay indicated miR‐1275 inhibited the activity of Wnt/β‐catenin pathway. **P < .01

In depth, we intended to figure out the probable targets that participated in the mechanism by which miR‐1275 modulated radiosensitivity and EMT of EC cells. And the RNA22 v2 (https://cm.jefferson.edu/rna22/Interactive/) predicted that WNT1, an activator of Wnt/β‐catenin signalling, was one of the potential downstream targets of miR‐1275. Additionally, we observed that the protein levels of β‐catenin and its targets including c‐myc and Slug were all aggrandized in radioresistant KYSE‐150R cells in comparison with the parental cells (Figure 3A). Also, miR‐1275 down‐regulation notably activated Wnt/β‐catenin signalling with boosted level of WNT1 and p‐GSK‐3β so as to reduce the level of p‐β‐catenin and enhance β‐catenin level; however, up‐regulation of miR‐1275 gave rise to observable inactivation of this pathway (Figure 3B), suggesting that miR‐1275 was a negative regulator of Wnt/β‐catenin signalling.

Afterwards, we made a further investigation into whether miR‐1275 affected Wnt/β‐catenin pathway through a WNT1‐mediated manner. Obviously, it was confirmed that WNT1 was directly targeted by miR‐1275 because only the luciferase activity of WNT1‐WT was strikingly strengthened under miR‐1275 inhibition but pronouncedly diminished upon miR‐1275 overexpression (Figure 3C,D). Moreover, the following RNA pull‐down assay and RIP assay further validated the interaction between miR‐1275 and WNT1 in a RNA‐induced silencing complex (RISC) (Figure 3E‐G). Furthermore, we observed that WNT1 was negatively regulated by miR‐1275 in EC cells (Figure 3H). Intriguingly, it was discovered that miR‐1275 mainly abolished the level of nuclear β‐catenin (Figure 3I). More importantly, the TOP/FOP ratio was sharply prompted in face of miR‐1275 down‐regulation but greatly abated in response to miR‐1275 up‐regulation (Figure 3J). Jointly, these findings demonstrated that miR‐1275 inactivated Wnt/β‐catenin signalling pathway in EC by targeting WNT1.

### 3.4 The role of miR‐1275 in EC radiosensitivity was dependent on WNT1

**Figure 4:**
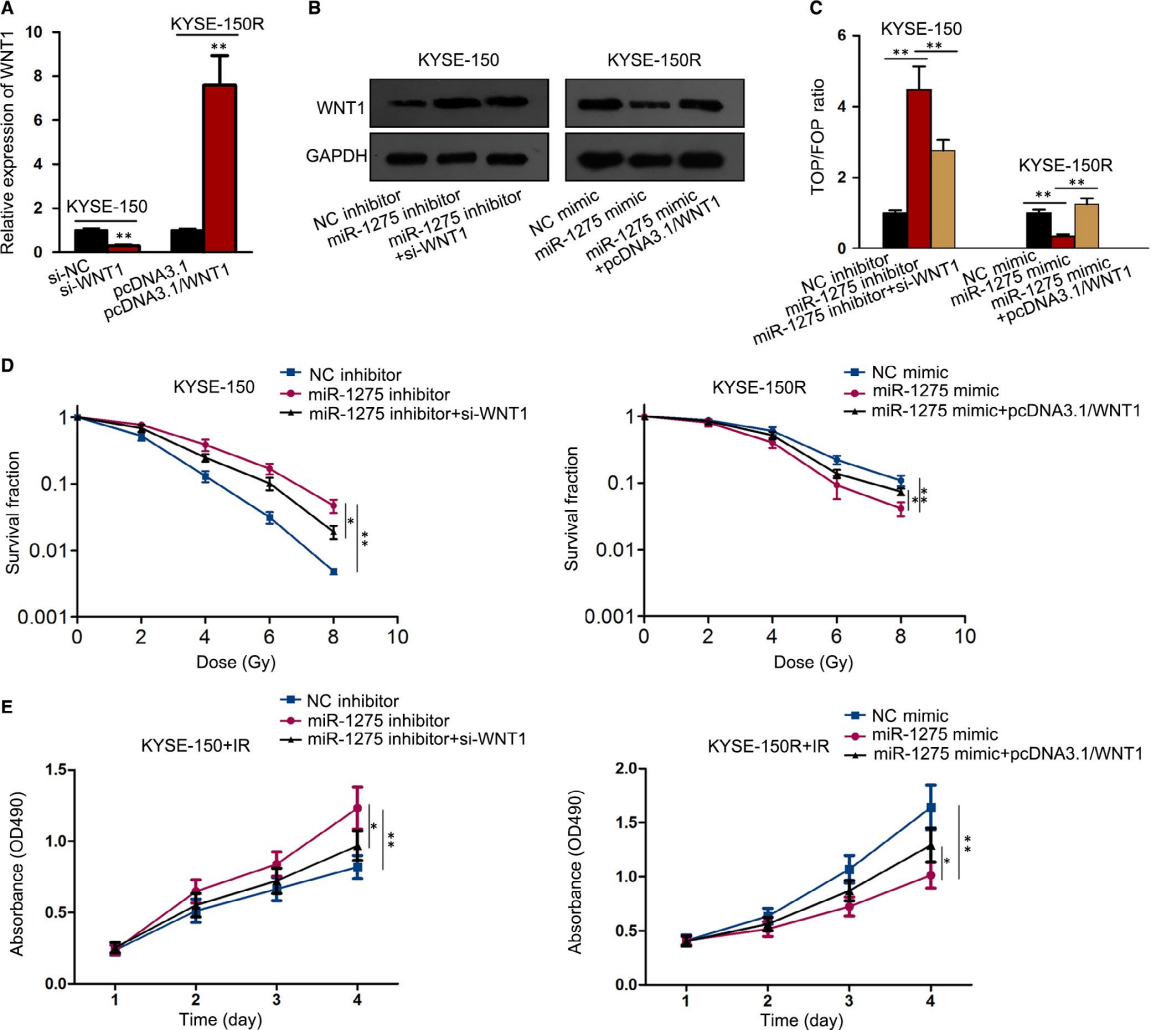
MiR‐1275 attenuated radioresistance of EC cells through targeting WNT1‐activated Wnt/β‐catenin pathway. A, Transfection efficiency of si‐NC or si‐WNT1 in KYSE‐150 cells and that of pcDNA3.1 or pcDNA3.1/WNT1 inKYSE‐150R cells transfected with was tested via qRT‐PCR. B, Protein levels of WNT1 in EC cells at different groups were determined by Western blot. C, TOP/FOP flash assay for the detection of Wnt/β‐catenin activity in various groups. D, The survival fraction of KYSE‐150 and KYSE‐150R cells under irradiation was assessed by colony formation assay. E, The cell viability of above cells was examined by MTT assay. *P < .05, **P < .01

Next, we studied whether WNT1 was implicated in miR‐1275‐affected EC radiosensitivity. Firstly, the expression of WNT1 was silenced in KYSE‐150 cells responding to si‐WNT1 transfection but up‐regulated in KYSE‐150R cells under the transfection of pcDNA3.1/WNT1, as indicated in Figure 4A. Besides, miR‐1275 inhibition‐stimulated WNT1 protein was normalized under WNT1 knockdown, whereas the protein level of WNT1 attenuated by miR‐1275 mimic was recovered upon WNT1 overexpression (Figure 4B). Also, the activity of Wnt/β‐catenin signalling influenced by miR‐1275 inhibition or stimulation was reversed, respectively, after WNT1 knockdown or overexpression (Figure 4C). Furthermore, WNT1 silence distinctly increased the miR‐1275 inhibition‐alleviated radiosensitivity in KYSE‐150 cells, whereas WNT1 up‐regulation overtly strengthened the miR‐1275 overexpression‐hindered radioresistance in KYSE‐150R cells (Figure 4D), with the SERs were 1.382 and 0.781, respectively. Meanwhile, though all cells exposed to radiation, the promoted viability of miR‐1275‐inhibited KYSE‐150 cells was abrogated in face of WNT1 depletion, whereas miR‐1275 overexpression‐impeded viability was recovered by enforced expression of WNT1 in KYSE‐150R cells (Figure 4E). Hence, we concluded that the radiosensitizing effect of miR‐1275 in EC was dependent on WNT1‐activated Wnt/β‐catenin pathway.

### 3.5 miR‐1275 had a suppressive impact on EMT in EC cells by a WNT1‐mediated way

**Figure 5:**
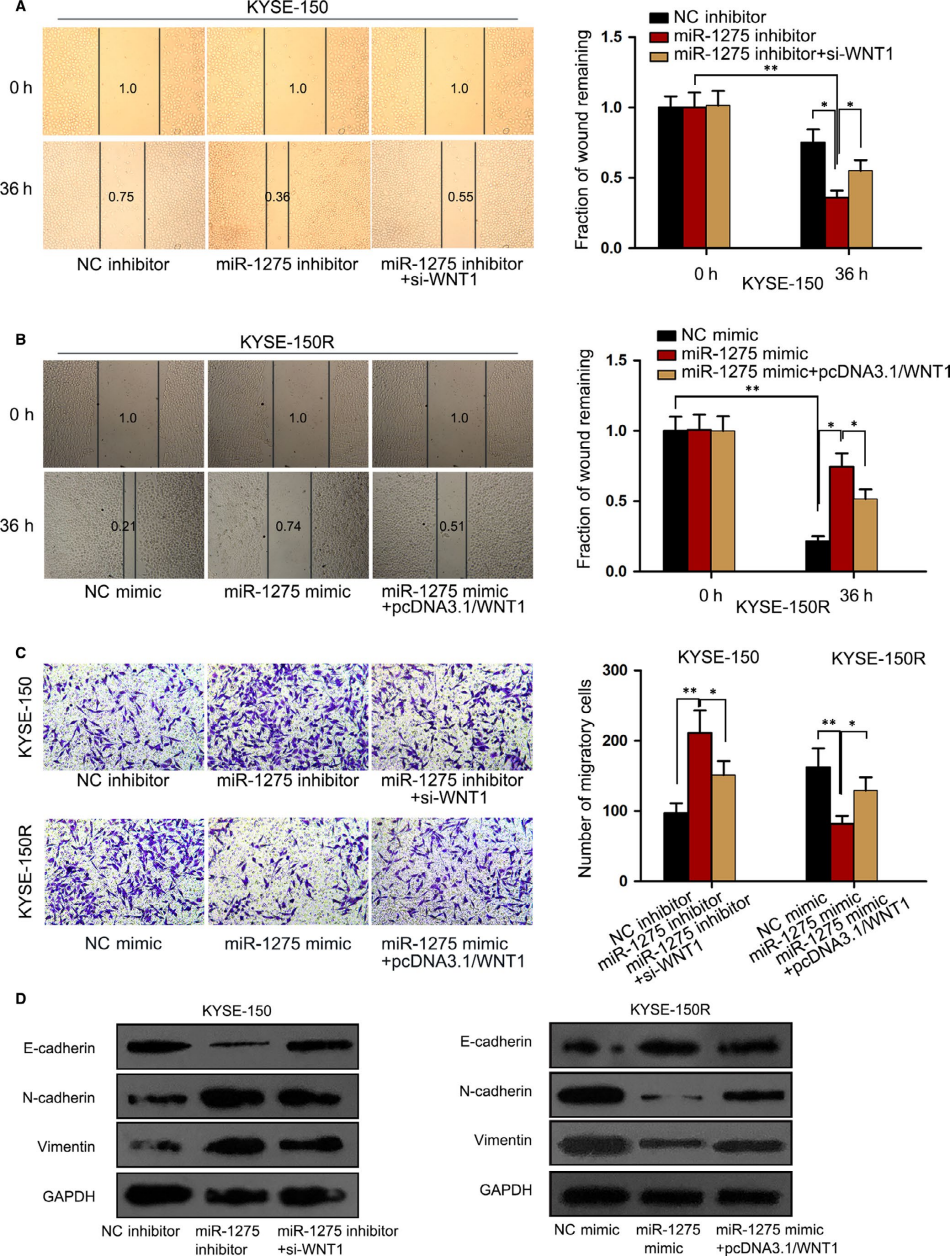
MiR‐1275 inhibited EMT in EC cells by abating WNT1 expression. KYSE‐150 cells were transfected with NC inhibitor, miR‐1275 inhibitor or miR‐1275 inhibitor together with si‐WNT1 and KYSE‐150R cells were transfected with NC mimic, miR‐1275 mimic or miR‐1275 mimic together with pcDNA3.1/WNT1. A, B, Wound‐healing assay was performed to evaluate cell migration in above KYSE‐150 cells and KYSE‐150R cells under different conditions. C, Transwell migration assay was also conducted in the above EC cells to assess cell migration. D, The level of EMT‐related proteins in indicated cells was detected by Western blot. *P < .05, **P < .01

Subsequently, we explored the impact of miR‐1275/WNT1 axis on the EMT process in EC cells. As demonstrated in Figure 5A, the remaining wound distance reduced by miR‐1275 inhibitor was partially reversed under co‐transfection of si‐WNT1. In contrast, ectopic expression of WNT1 offset the inhibition of miR‐1275 up‐regulation on EC cell migration (Figure 5B). Conformably, the results of Transwell assay represented a similar outcome that cell migration affected by miR‐1275 inhibition or stimulation was recovered in the context of WNT1 silence or overexpression, respectively (Figure 5C). Moreover, the results of Western blot analysis also stated that silencing WNT1 normalized the miR‐1275 inhibition‐induced EMT in KYSE‐150 cells while overexpressing WNT1 rescued the miR‐1275 up‐regulation‐hampered EMT in KYSE‐150R cells, verifying that the negative modulation of miR‐1275 on EMT was mediated by WNT1 (Figure 5D). By and large, miR‐1275 enhanced radiosensitivity in EC cells due to its impairment on EMT via a WNT1‐mediated pathway.

### 3.6 MiR‐1275 confined tumour growth and prompted EC rdiosensitivity in vivo

**Figure 6:**
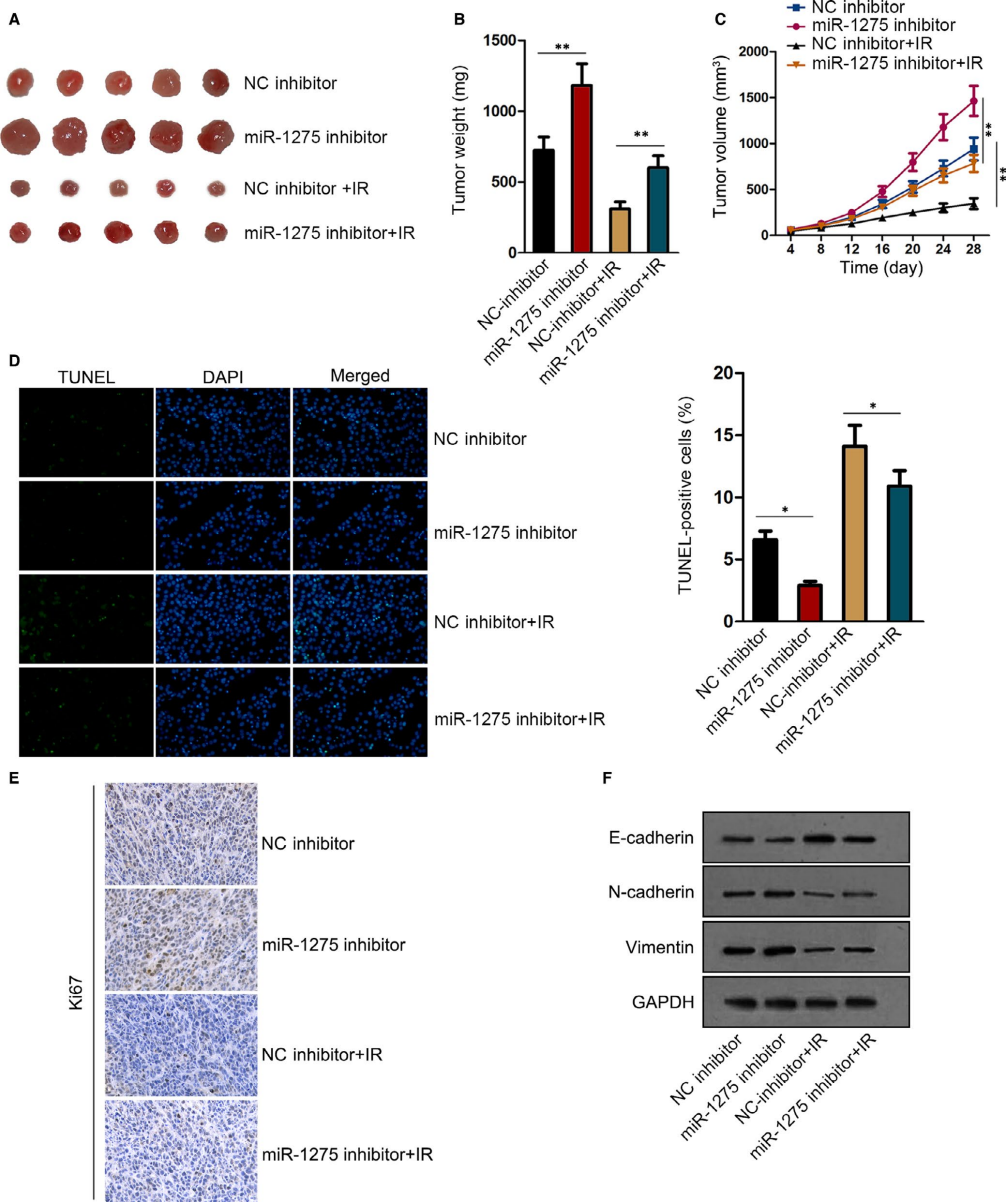
Inhibition of miR‐1275 promoted the growth and the radioresistance of EC cells in vivo. A, Representative images of tumours from mice inoculated with KYSE‐150 cells transfected with miR‐1275 inhibitor or NC inhibitor, as well as the images of those from mice with the same inoculation but together with IR exposure. B, C, The weight (B) and size (C) of tumours from above four groups. D, E, The cell apoptosis and proliferation of tumours from four groups were, respectively, estimated by TUNEL assay (D) and IHC staining of Ki67 (E). F, The level of E‐cadherin, N‐cadherin and Vimentin in above xenografts was assessed via Western blot. *P < .05, **P < .01

To further confirm the part of miR‐1275 in EC tumour growth and radiosensitivity, the in vivo experiments were carried out through inoculating the KYSE‐150 cells with or without miR‐1275 inhibition into naked mice (five mice in each group). Seemingly, miR‐1275 inhibition markedly accelerated while IR exposure obviously restrained the growth of tumours; more importantly, the tumours derived from miR‐1275‐silenced cells were still bigger and heavier than those originated from control cells, despite both were treated with IR (Figure 6A‐C). In addition, cell apoptosis was abrogated under miR‐1275 suppression but encouraged by IR treatment, and inhibition of miR‐1275 had a mitigatory effect on the apoptosis of EC cells facing exposure (Figure 6D). Conversely, the Ki67 staining in KYSE‐150 cells represented as an absolutely opposite phenomenon to above results when under the same conditions (Figure 6E). Furthermore, we validated that EMT was enhanced by miR‐1275 silence but obstructed under IR treatment, with a promoted EMT in irradiated KYSE‐150 cells in response to miR‐1275 suppression (Figure 6F), indicating that miR‐1275 stimulated EC radiosensitivity by inhibiting EMT in vivo. On the whole, miR‐1275 exerted an inhibitory function in EC cell growth and it strengthened the sensitivity of tumours to radiation by suppressing EMT in vivo.

## 4 DISCUSSION

Over the past decades, the emerging role of ncRNAs, including miRNAs, have been reported in the development of EC radioresistance.^28^, ^29^, ^30^, ^31^ In this study, we proved that miR‐1275, which has been revealed to exert paradoxical functions in different cancer types,^12^, ^32^, ^33^, ^34^ was down‐regulated in radioresistant EC cells. Meanwhile, we illustrated that miR‐1275 could increase the sensitivity of EC cells to radiotherapy both in vitro and in vivo. Intriguingly, the radiosensitization of miR‐1275 in EC was demonstrated to be attributed to the negative regulation of miR‐1275 on EC cell migration and EMT, while the association between EMT and radioresistance has already been uncovered by previous studies.^27^, ^35^

Next, we attempted to explore the in‐depth mechanism whereby miR‐1275 inhibited EMT and therefore enhanced radiosensitivity. Wnt/β‐catenin signalling pathway is a well‐recognized oncogenic pathway that is involved in a multitude of biological processes, including cell proliferation, differentiation, migration, genetic stability and apoptosis,^36^ and the aberrant regulation of this pathway usually led to tumorigenesis of multiple cancers.^37^ Furtherly, recent researches have highlighted the participation of Wnt/β‐catenin pathway not only in cancer progression^37^, ^38^ but also in the development of radiation resistance in numerous carcinomas.^39^, ^40^ Also, the interplay between miRNAs and Wnt/β‐catenin pathway in regulating EMT has also been indicated before.^41^ Presently, we discovered this pathway also mediated the influence of miR‐1275 on EC cell radioresistance.

Fortunately, WNT1, a key molecule involved in Wnt/β‐catenin pathway,^42^ was predicted and verified as the direct target of miR‐1275 for the first time in this study, so that miR‐1275 negatively regulated Wnt/β‐catenin signalling in EC cells by targeting WNT1. Collectively, we proved that miR‐1275 affected EC radiosensitivity by targeting WNT1‐activated Wnt/β‐catenin pathway, and similar mechanism was previously unearthed in the influence of miR‐301a on the migration and radiosensitivity of EC cells.^43^ More importantly, the rescue of WNT1 on miR‐1275‐regulated EC radiosensitivity was mediated by the enhancement of WNT1‐regulated Wnt/β‐catenin signalling per se on EMT,^44^, ^45^ thus indicating the strong association among miR‐1275, EMT and WNT1 in regulating the radiosensitization of EC cells.

In current study, we disclosed unsurprisingly that miR‐1275 reduced EC cell radioresistance through impairing EMT via inactivating Wnt/β‐catenin pathway by targeting WNT1, indicating a new effective target to overcome radioresistance of EC patients. However, to intensify the clinical significance of miR‐1275 as a therapeutic target for radioresistant EC patients, the effect of reagents or drugs that result in miR‐1275 up‐regulation needs to be further assayed in advanced animals and even human in the future.

## CONFLICT OF INTEREST

None.

## AUTHOR CONTRIBUTIONS

Congying Xie contributed to the design of the study. Youyi Wu wrote the manuscript. Zhenghua Fei, Congying Xie and Huafang Su performed the experiments. Ya Fang and Shenlan Xiao analysed the data. All authors read and approved the final manuscript.

## ETHICAL APPROVAL

In vivo experiment was performed under the experimental animal use guidelines of the ethics committee of The First Affiliated Hospital of Wenzhou Medical University.

## Data Availability

Research data and material are not shared.
