# Correction to “MicroRNA‐1275 induces radiosensitization in oesophageal cancer by regulating epithelial‐to‐mesenchymal transition via Wnt/β‐catenin pathway”

**DOI:** 10.1111/jcmm.70706

**Published:** 2025-07-07

Xie C, Wu Y, Fei Z, Fang Y, Xiao S, Su H. MicroRNA‐1275 induces radiosensitization in oesophageal cancer by regulating epithelial‐to‐mesenchymal transition via Wnt/β‐catenin pathway. J Cell Mol Med. 2020 Jan; 24(1):747–759. doi: https://doi.org/10.1111/jcmm.14784.

In Xie et al., the images for western blotting of β‐catenin and GAPDH in Figure 3I were used incorrectly due to a technical error during image preparation. The correct figure is shown below. The authors confirm that all results and conclusions of the article remain unchanged.

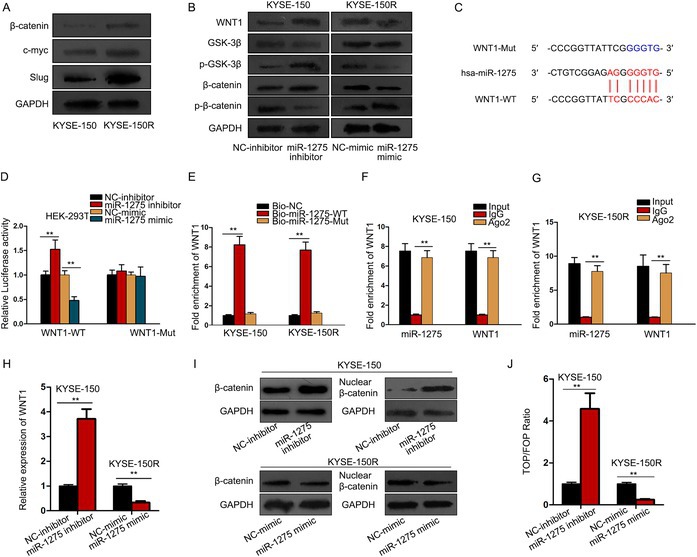

MiR‐1275 inactivated Wnt/β‐catenin pathway in EC cells through directly targeting WNT1. (A) Western blot results of the protein level of β‐catenin and its targets in KYSE‐150 and KYSE‐150R cells. (B) The protein levels of the members involved in Wnt/β‐catenin signalling in miR‐1275‐inhibited KYSE‐150 cells and miR‐1275‐up‐regulated KYSE‐150R cells were examined by Western blot. (C) Predicted binding sites between miR‐1275 and WNT1, as well as the sequences of WNT1‐Mut with mutated binding sites in 3′‐UTR of WNT1. (D) Luciferase reporter assays for the luciferase activity of WNT1‐WT and WNT1‐MUT in HEK‐293T cells with different transfections. (E–G) RNA pull‐down assay (E) and RIP experiments (F, G) were conducted in indicated EC cells for further confirmation of the interaction between miR‐1275 and WNT1. (H) The effect of miR‐1275 on the expression of WNT1 in EC cells was assessed by qRT‐PCR. (I) The level of total β‐catenin and nuclear β‐catenin in KYSE‐150 cells with miR‐1275 inhibition and KYSE‐150R cells with ectopic expression of miR‐1275 was detected via Western blot. (J) TOP/FOP flash assay indicated miR‐1275 inhibited the activity of Wnt/β‐catenin pathway. **p < 0.01.

